# Problematic Smartphone Use—Comparison of Students With and Without Problematic Smartphone Use in Light of Personality

**DOI:** 10.3389/fpsyt.2020.599241

**Published:** 2021-01-28

**Authors:** Christiane Eichenberg, Markus Schott, Athina Schroiff

**Affiliations:** ^1^Fakultät für Medizin, Sigmund Freud PrivatUniversität Wien, Wien, Austria; ^2^Technische Universität München, München, Germany; ^3^Fakultät für Psychologie, Sigmund Freud PrivatUniversität Wien, Wien, Austria

**Keywords:** smartphone, internet, addiction, personality, online

## Abstract

**Background:** As a device with multiple functions, a smartphone become more and more relevant in everyday life. However, this goes along with an increase in reports about smartphone addiction and its unwanted consequences. One of the most important variables in the etiopathogenesis of addictive smartphone use is personality.

**Objective:** This study aimed to investigate predictors of problematic smartphone use. Clinically relevant differences in personality, psychopathology, and social support between students with and without problematic smartphone use were investigated.

**Method:** All currently enrolled students at the Sigmund Freud University in Vienna (*N* = 1,836) were surveyed. Response rate was 27.07% (*N* = 497, age: M = 19.6, SD = 8.04). The Smartphone Addiction Scale (SPAS), the 10-Item Big Five Inventory (BFI-10), the Brief Symptom Inventory (BSI-18), and a questionnaire on social support (F-SozU-K-14) were used.

**Results:** A total of 75 students (15.1% of the total sample) showed problematic smartphone use. In terms of personality, respondents with problematic smartphone use showed significantly higher values for extraversion and neuroticism compared than non-addicted users. Students with problematic smartphone use showed significantly higher levels in terms of depression and anxiety. Contrary to expectations, individuals with problematic smartphone use showed significantly higher values for perceived social support than with individuals without problematic smartphone use.

**Discussion:** Therapy for problematic smartphone use should be carried out taking into account discussed, important etiological factors, such as personality.

## Introduction

Smartphones have become an essential part of everyday life. In western societies, almost all adolescents (98%) own a smartphone (1). Research suggests that on average adolescents interact with digital media (i.e., watching videos, reading news, or using social media) for more than 6.5 h every day; mobile devices account for nearly half this time (2). Teens aged 18–24 years look at their smartphone an average of 214 times each day (3). Therefore, it is not surprising that literature increasingly finds people to use their phones in ways that may cause problems with their health (4). There are not only problematic physical effects, such as neck pain (5) or accidents affecting pedestrians (6), but also mental health problems, including sleep disturbances, depression, problems with interpersonal relationships (7), and even smartphone addiction (8).

Currently, smartphone addiction has not been mentioned in either the *Diagnostic and Statistical Manual of Mental Disorders* [DSM-5; (9)] or in the *International Classification of Diseases*, 11th Revision [ICD-11; (10)]. “Gambling disorder” and its specific variant the “Internet gaming disorder” are the only non-substance-related disorders included in the DSM-5 (9). Nonetheless, other potential online addictions and Internet use-related disorders have been reviewed. The introduction of the new diagnoses “gaming disorder” and other Internet-related disorders in the upcoming ICD-11 under the category associated with addictive disorders (“Disorders due to addictive behaviors”) reflects the importance of media-related disorders for current research and clinic. In addition, a disorder category “hazardous gaming” will be introduced under the cluster “factors associated with health behaviors” in order to address media-related problem behavior diagnostically and avoid future addiction. This indicates that limiting diagnosis to online gaming exclusively may disregard many other Internet-related behaviors that can be engaged in addictively (11). In the literature, it is argued that to not overlook individuals who suffer from considerable impairments as a consequence of their problematic Internet use, until the concept is grasped more comprehensively, research on Internet addiction should not be limited by only focusing on one aspect of media addiction. Consequently, the diagnosis “Internet addiction” is frequently understood more broadly (12). The core diagnostic characteristics of the Internet addiction consist of mental preoccupation with the Internet, development of tolerance, social withdrawal, frustrations with relapse, withdrawal symptoms (irritability, anxiety, and sadness), loss of interest in previous hobbies or activities, continuation of problematic consumption despite the knowledge of the resulting psychosocial problems, dysfunctional affect regulation, and lying to friends, family members, or therapists to conceal actual consumption as well as the loss of a meaningful relationship, job or apprenticeship, or career opportunities (9, 13–15).

As smartphones offer a wide array of possibilities and functions to access the Internet, Elhai et al. (16) highlight that Internet and smartphone addiction are closely related constructs and share much communality. In line with Biang and Leung (17), characteristics of problematic smartphone use are comparable with the diagnostic criteria of an Internet addiction. Similarly, Lin et al. (18) demonstrated that smartphone addiction shares the main diagnostic factors with other DSM-5 substance and non-substance disorders, proposing the following diagnostic criteria for smartphone addiction: compulsive behavior, functional impairment, withdrawal, and tolerance (19). To date, few studies have investigated problematic smartphone use. Existing studies have focused on prevalence rates (20), diagnostic criteria (19), development, and validation of instruments designed to assess smartphone addiction (18, 21, 22). Little is known about indicators and etiopathogenetic factors of problematic smartphone use. A recent study by Eichenberg et al. (23) investigated attachment-specific differences between smartphone-addicted and non-addicted individuals. Overall, the assumption that insecure people more often showed problematic smartphone use was confirmed; especially “ambivalent-closed” attachment styles were related with problematic smartphone use. However, the authors underline the necessity to identify additional predictors that might promote the development of problematic smartphone use (23).

Another particularly important risk factor seems to be psychopathology (24). Available research indicates that depression or anxiety might lead to media addiction. For instance, stressed and depressed individuals use online video gaming as a coping mechanism to relieve depressive and negative emotions (25). This association was confirmed in a systematic review by Elhai et al. (16), showing that both depression and anxiety severity were consistently linked with problematic smartphone use. An important theoretical consideration is whether smartphone addiction is related to the smartphone itself or if the smartphone is just a medium through which individuals access other addictions. Smartphones provide many possibilities and functions that even further increase the likelihood to develop obsessive behaviors (26). Particularly, social aspects, i.e., social networks, such as Facebook, Instagram, and Snapchat, play a considerable role in facilitating the smartphones' addictive potential. This idea is supported by research showing that social network use and gaming play a major role in addictive smartphone use (27). Taking into account these social functionalities that smartphones offer, users' perceived social support can be regarded as a major predictor of smartphone use. It can be argued that the need to obtain social support in users with low social support can increase the potential risk of smartphone addiction (28). On the other side, it seems reasonable that problematic smartphone use can socially isolate individuals and lower their social support.

A widely agreed on etiopathogenetic factor associated with problematic media consumption is personality. One of the most notable personality theories is the five-factor model of personality, which sets apart five main dimensions: “neuroticism” (e.g., being nervous and anxiety prone), “extroversion” (e.g., being talkative and outgoing), “openness to experience” (e.g., being imaginative and intellectually oriented), “agreeableness” (e.g., being sympathetic and warm), and “conscientiousness” (e.g., being organized and prompt) (29). These traits have been validated across most cultures (30). Andreassen et al. (31) carried out one of the first surveys identifying the inter-relationship between the “Big Five” personality traits and behavioral addictions. Whereas, neuroticism was positively related to Internet addiction, agreeableness and conscientiousness were negatively related to Internet addiction. Kayis et al. (32) showed in a meta-analysis that all Big Five personality traits had a significant correlation with Internet addiction, whereas openness to new experiences, conscientiousness, agreeableness, and extraversion were negatively associated with Internet addiction neuroticism and showed a positive association with it. Similarly, Kuss et al. (33) confirmed neuroticism and low agreeableness to be related with Internet addiction. Overall, neuroticism has been associated with Internet addiction or problematic Internet use in several studies (34, 35).

Given the discussed similarities across Internet and smartphone addiction, it is likely that personality traits might also be related to smartphone addiction (36, 37). In this sense, Van Deursen et al. (38) revealed that poor self-control may be a cause of smartphone addiction. Also, shy, lonely, and depressed respondents were prone to a Smartphone addiction (39). In a research project by Hussain et al. (40), a significant association between smartphone addiction and emotional instability was established. In a mixed-method study, narcissistic personality disorder was found to be a risk factor for developing a smartphone addiction (41). Similarly to findings regarding Internet addiction, a very recent publication by Lei et al. (42) has found a poor positive correlation between smartphone addiction and neuroticism. Unfortunately, most studies on personality and problematic smartphone use focus on only some Big Five personality traits, especially neuroticism [e.g., (43)]. In their meta-analysis, Marengo et al. (44) also report an association between conscientiousness, agreeableness, and neuroticism and problematic smartphone use. Time of publication was related with increased strength of the correlation. In this sense, the current studies reported stronger effects than older publications. However, authors strongly highlight existing limitations in the current literature. For example, available data are dated, and the very limited number of studies at hand lowers the meta-analysis' ability to detect small-sized moderation effects, leading to low statistical power (44). A majority of included studies were carried out in English-speaking countries, underlying the need for increased diversity in this research area, especially considering cultural differences in personality traits.

With the growing amount of time that individuals spend with online media, this mentioned scarcity of research investigating smartphone addiction is rather surprising. Therefore, considering the discussed research, the aim of this study was to examine whether certain personality traits are related to problematic smartphone use. Additionally, interrelationships between psychopathology and social support were investigated.

It was hypothesized that there is a positive relationship between extraversion, neuroticism, psychopathology (i.e., depressive symptoms and anxiety), and problematic smartphone use. It was further hypothesized that there is a negative relationship between social support and problematic smartphone use.

## Method

### Study Design

All currently enrolled students at the Sigmund Freud University in Vienna (*N* = 1,836) were surveyed. Participation was voluntary, and no extra university credits were offered. Data were collected with the online survey tool Unipark. A pre-test was carried out with nine participants. Returns were evaluated, and the survey was modified regarding its practicability, comprehensibility, and completeness of item formulation. Data collection took place starting from 17 March 2017 and ending on 13 May 2017. General information about purposes of the study, study design, and confidentiality was given to participants. Altogether, the online study was accessed 843 times. Most dropouts happened on the first page (23%); after the second page, only roughly 8% of respondents discontinued the survey. Consequently, the overall dropout rate of 59.96% is tolerable. Overall, 497 complete records were submitted. It took about 15 min to complete the survey. The Sigmund Freud University Vienna ethics committee granted ethical approval. For data input, processing, and statistical analyses, the Statistical Package for Social Sciences Program (SPSS Version 24) was used. Significance was checked using Whitney *U*-tests since prerequisites for performing a *T*-test (no normal distribution of variables in the two groups) were not met.

### Material

Sociodemographic data on age, gender, nationality of participants, and field of study were collected. Subsequently, participants reported on their time spent using the smartphone and preferred smartphone services. Four categories were available: research, entertainment (gaming, music, and reading), social media (SMS and calls), and utilities (photos, videos). Categories were rated on a 5-point-Likert scale (“never” to “daily”). In addition, participants were asked to fill out the following questionnaires.

#### Smartphone Addiction Scale

To assess symptoms of smartphone addiction, the Smartphone Addiction Scale (SPAS) by Biang and Leung (17) was used. This instrument is based on three separate inventories: the Mobile Phone Problem Use Scale [MPPUS-10; (45)], the Internet Addiction Test (46), and the Television Addiction Scale (47). The questionnaire consists of 19 items. A reliability coefficient of 0.70 is reported. The Cronbach α is high at 0.92.

Within this instrument, Young's (46) eight classic criteria of Internet addiction similar to those embedded in DSM-IV for diagnosing gambling-related problems were used to create the composite smartphone addiction index (SPAI). Sample items are “You have tried to hide from others how much time you spend on your smartphone” and “You find yourself engaged on the smartphone for longer periods of time than intended.” These eight items were later also used by Leung (48) to develop a scale for assessing addictive mobile phone use. As in this study it was needed to only differentiate between participants with and without problematic smartphone, only these eight items were applied.

Five primary factors are assessed: ignoring harmful consequences, excessive thinking about using the smartphone, inability to control desire, loss of productivity, and anxiety (17).

Equivalent to the method of Young (46), the 5-point Likert scale was dichotomized. Finally, answers were summed up, resulting in total scores ranging from 0 to 8. Consistent with data by Young (46) on Internet addiction participants scoring, more than five were diagnosed with problematic smartphone use.

#### 10-Item Big Five Inventory

The 10-Item Big Five Inventory [BFI-10; (49)] is a short-scale version of the well-established BFI. Only two items per dimension are assessed. This instrument is made up of 10 items rated on a 5-point Likert scale (1 = “completely disagree” and 5 = “completely agree”). Research underlines that the BFI-10 has psychometric properties comparable in size and structure to those of the full-scale BFI. Mean retest stability coefficients were.75. All five subscales show a satisfactory to good retest reliability, ranging from 0.58 for agreeability to 0.84 for extraversion. Factorial validity was confirmed using a confirmatory factor analysis. On the basis of the factor loadings, acceptable validity could be determined. Convergent validity correlations with the NEO-PI-R domain scales averaged 0.67, showing substantial convergent and discriminant validity (49).

#### Short Version the Brief Symptom Inventory (BSI-18)

The BSI-18, the short version of the Brief Symptom Inventory, comprises 3 six-item scales (somatization, depression, and anxiety) and a global parameter. All scales show good reliability coefficients [Cronbach's α = 0.79 for somatization, 0.84 for depression, 0.84 for anxiety, and 0.91 for Global Severity Index (GSI)]. The postulated three-factor structure could be confirmed with both exploratory and confirmatory factor analyses. The questionnaire separates sufficiently between different patient groups. External assessment by therapists correlated well with the self-assessment. In summary, psychometric values of the BSI-18 are satisfactory. Compared with the full Symptom Checklist (SCL-90-R), loss of information due to the reduction to 18 items is acceptable in the analysis of large samples (50).

#### Short version of the Social Support Questionnaire

The Short version of the Social Support Questionnaire (F-SozU-K-14) is a 14-item questionnaire to assess perceived and anticipated social support. Items are rated on a 5-point Likert scale (1 = “does not apply at all” to 5 = “totally applies”). The inventory is characterized by very good item statistics as well as good internal consistency (Cronbach's α = 0.94). Correlations with sociodemographic variables and correlations with external criteria were checked for validity of the Social Support Questionnaire. Taken together, both internal consistency and other psychometric properties can be rated as very satisfactory (51). The final value is determined by summing the item responses and then dividing by the number of items answered, whereby higher values indicate higher social support.

### Sample

The final sample consisted of *N* = 497 participants (*n* = 120 men (24.2%) and *n* = 377 women (75.8%). Subjects were mainly from Germany (72.8%) and Austria (13.6%), and just 3% had another nationality. No data on nationality were available for 10.6% of participants. The age range was from 17 to 70 years (M = 19.38 years, SD = 16.50). A majority of participants were students of psychotherapy (*n* = 286, 57.5%) or psychology (*n* = 125, 25.2%); 16.5% were enrolled in medicine (*n* = 82) and 0.6% in law (*n* = 4). In light of the composition of currently enrolled students at the Sigmund Freud University, this distribution was expected.

## Results

### Personality, Psychopathology, and Social Support

Regarding personality, participants scored the highest on openness (M = 3.76, SD = 0.94) and conscientiousness (M = 3.47, SD = 89) and the lowest on neuroticism (M = 2.99, SD = 0.99). In terms of psychopathology study, participants scored relatively high (depression M = 9.31, SD = 3.96; anxiety M = 9.34, SD = 3.16) than a clinical sample [depression scores for a sample of patients with depression (M = 11.64, SD = 6.09) and anxiety (M = 7.90, SD = 5.61)] (50). Overall, participants report high values on social support (M = 4.45, SD = 0.63).

Table 1 presents mean scores and standard deviations for the following study variables: psychopathology (depression, anxiety), personality (extraversion, agreeableness, conscientiousness, neuroticism, and openness), and social support.

**Table 1:** Mean scores and standard deviations for study variables.

	**Mean (M)**	**Standard deviation (SD)**
**Personality**		
Extraversion	3.45	0.98
Agreeableness	3.23	0.83
Conscientiousness	3.47	0.89
Neuroticism	2.99	0.99
Openness	3.76	0.94
**Psychopathology**	26.58	8.1
Depression	9.31	3.96
Anxiety	9.34	3.16
Social support	4.45	0.63

### Problematic Smartphone Use

For a comprehensive analysis, essential data were missing for *n* = 19 subjects (1.4%). Problematic smartphone use was diagnosed in *n* = 75 (15.1%) of participants, 86.7% were female, and a minority (13.3%) were male. Yet this distribution is comparable with the gender ratio of the study sample.

Presented services were used roughly for the same amount of time. The most important smartphone service was “communication” (M = 4.9, SD = 0.5). The service “entertainment” (M = 4.4, SD = 1.02) was used least frequently. For information research and other utilities, smartphones were used comparably often (M = 4.6, SD = 0.77).

### Problematic Smartphone Use and Personality, Psychopathology, and Social Support

For a comprehensive overview of differences between users with and without problematic smartphone use for the variables personality, psychopathology, and social support, see Table 2.

**Table 2:** Results of Mann–Whitney *U*-tests for personality, psychopathology, social support, and problematic smartphone use (significant differences are highlighted).

**Problematic smartphone use and**	***U***	***Z***	***p***
**Personality**			
Extraversion	13,021.00	2.28	**0.023**
Agreeableness	14,564.5	−0.902	0.364
Conscientiousness	13,604.5	−1.731	0.084
Neuroticism	11,763.00	3.35	**0.001**
Openness	14,890.5	−0.538	0.590
**Psychopathology**			
Depression	10,322.50	−4.70	**<0.001**
Anxiety	11,118.50	−3.98	**<0.001**
Social support	12,604.00	−2.63	**0.009**

#### Problematic Smartphone Use and Personality

A significant difference between users with and without problematic smartphone use in light of extraversion (*U* = 13,021, 00, *Z* = −2.28, *p* = 0.023) was found. Participants with problematic smartphone use (M = 3.71, SD = 0.92) were found to have higher scores on extraversion than users without problematic use (M = 3.39, SD = 0.99). Similarly, groups differed significantly considering neuroticism (*U* = 11,763.00, *Z* = −3.35 *p* = 0.001). Users with problematic smartphone use showed higher levels of neuroticism (M = 3.35, SD = 0.88) than non-dependent users (M = 2.93, SD = 1.00).

As expected, considering the personality factors agreeableness (*U* = 14,564.5, *Z* = −0.902, *p* = 0.364), openness (*U* = 14,890.5, *Z* = −0.538, *p* = 0.590), and conscientiousness (*U* = 13,604.5, *Z* = −1.731, *p* = 0.084), no significant differences between groups were found.

#### Problematic Smartphone Use and Social Support

A significant difference between study respondents with and without problematic smartphone use behavior and perceived social support was found (*U* = 12,604.00, *Z* = −2.63, *p* = 0.009).

Surprisingly, users with problematic smartphone use (M = 4.59, SD = 0.66) indicated higher perceived social support than users without problematic smartphone use (M = 4.44, SD = 0.59).

#### Problematic Smartphone Use and Psychopathology

Further analyses revealed a significant difference between groups for depressive symptoms (*U* = 10,322.5, *Z* = −4.70, *p* ≥ 0.001). Participants with problematic smartphone use (M = 11.27, SD = 4.71) had higher depression scores than users without problematic smartphone use (M = 8.99, SD = 3.72).

Finally, a significant difference between users with and without problematic smartphone regarding anxiety (*U* = 11,118.5, *Z* = −3.98, *p* ≥ 0.001) was found. Users with problematic smartphone use (M = 10.71, SD = 3.59) showed higher anxiety levels than users without problematic smartphone use (M = 9.13, SD = 3.03).

### Bonferroni–Holm Corrections

The Bonferroni–Holm method was used to counteract the problem of multiple comparisons in this study and control the family-wise error rate. All significant findings could be confirmed using the Bonferroni–Holm method (Table 3).

**Table 3:** Bonferroni–Holm corrections for study hypotheses.

**Comparison** **(problematic smartphone use)**	***p***	**Adjusted Bonferroni–** **Holm p**	**Significance**
Extraversion	*p* = 0.023	α = 0.050	Significant
Neuroticism	*p* = 0.001	α = 0.010	Significant
Depression	*p* < 0.001	α = 0.007	Significant
Anxiety	*p* < 0.001	α = 0.008	Significant
Social Support	*p* = 0.009	α = 0.016	Significant

## Discussion

Despite an abundance of evidence showing the relationship between personality traits and addictive behavior (32), the scientific literature is scarce in studies emphasizing the interconnections between Big Five personality traits and problematic smartphone use (44). There are some findings showing that psychopathology is associated with problematic use of technology (52, 53). The purpose of this study was to replicate and add to previous studies and fill in knowledge gaps particular in regard to problematic smartphone use.

In the present study, 15.1% of respondents were diagnosed with problematic smartphone use. This prevalence rate is in line with other current research (41, 54).

Neuroticism and extraversion were found to be positively related to problematic smartphone use. Neuroticism most probably reflects underlying traits of social interaction-based anxiety, fear of failure, or a firm superego [Kets (55)]. It has been proposed that due to social insecurity, communicating online is favored to offline communication by individuals with high scores on neuroticism (37). Therefore, one explanation for this finding might be that problematic smartphone use might be a way of escaping from social anxiety (31). Similarly, virtual communication might allow for easier interpersonal exchange as a punitive superego might be attenuated. Extroverted individuals are assumed to frequently seek out stimulation and consequently being more prone to addictive behaviors (56). This is consistent with the theory proposed by Bianchi and Philips (45) that the facet excitement-seeking of the dimension extraversion can be regarded as a risk factor for problematic smartphone use.

No relationship was found for agreeableness, openness, and conscientiousness and problematic smartphone use. These results are similar to those of Trub and Barbot (57) and Volungis et al. (58), who found no associations between these personality traits and Smartphone addiction. However, older research by Hwang and Jeong (59) even showed a positive association. This might be due to smartphones no longer representing novel products. Therefore, the interest of open-minded individuals might decrease and consequently the association between openness and problematic smartphone use. In line with the notion that people high on agreeableness scores are likable and pleasant and emphasize harmony in relationships, this personality trait might even be a protective factor against problematic smartphone use (60). Similarly, conscientious people are described as organized and hardworking and therefore might be less susceptible to problematic smartphone use.

Study findings indicate that problematic smartphone use commonly co-occurred with psychopathology, i.e., depression and anxiety. There is literature suggesting that chronically stressed individuals use smartphones as a coping mechanism to relieve depressive symptoms (16). In this sense, smartphone use might serve as an avoidance strategy to avoid negative emotions—even though being ineffective and even having adverse emotional consequences (61). On the other hand, there is research showing that higher levels of technology use go along with psychopathology (7). This might be due to problematic smartphone use keeping users awake at night (8) or increasing work demands, for instance, working from home or being available even after work hours (62). In conclusion, there might be a bidirectional association whereby problematic smartphone use drives psychopathology and vice versa (16). This mutual influence of symptom severity (media use and depression score) became clear in a longitudinal study by Gentile (63). Interestingly, as addictive digital media use increased or decreased, so did depressive symptoms.

In the present study, participants with problematic smartphone use reported higher perceived levels of social support than users without problematic smartphone use. This can be interpreted as individuals with problematic smartphone use dysfunctionally attribute smartphone use as effective and maybe their only means to establish and receive social support, while in fact their non-face-to-face communication might make them feel even more lonely and isolated (64). Alternatively, it can be argued that smartphone use actually goes along with more perceived social support and a feeling of being connected with others. In the digital era, the concept of social support needs to be critically reviewed. What is regarded as social support needs to be reevaluated. “Likes” on Facebook or Instagram might be experienced as actual social reciprocity.

## Limitations

This study needs to be interpreted with caution due to methodological limitations. First of all, the sample was drawn from a specific population, and an even smaller subset completed the study. Subjects were mainly enrolled in psychology/psychotherapy courses at Sigmund Freud University. Little is known about individuals who did not complete the study. This selection bias is inherent to web-based surveys, and the self-selection process limits the validity of findings. For instance, smartphone users who are interested in relativizing the negative image of technology addiction might be more encouraged to participate. Additionally, age distribution was very narrow, and female participants contributed disproportionately to study data. This gender bias in web-based studies has commonly been reported (65). Future studies need wider recruitment methods to produce more generalizable data. As a retrospective study, the possibility of memory biases needs to be discussed (66). Memory gaps might have been replaced with previously anchored schemata. Therefore, the validity of the study might be affected. Nonetheless, the risk of memory distortions can be regarded as insignificant considering that at the time of the study respondents were actively using smartphones on a daily basis. Therefore, study participants' responses can be viewed as a reliable source of data. Further, it can be argued that data on smartphone usage and preferred services are somewhat unreliable, as they were gathered from participants' self-reports. However, these self-reports were only used for descriptive measures; no further statistical analyses were conducted with these data. Therefore, this form of collecting data can be regarded as sufficient.

## Conclusion

Overall, current findings add to previous reports in several ways. First of all, up-to-date findings fill the surprising lack of current data in this research area (44). This is especially important, considering that the association between personality traits and smartphone use might change over time (i.e., discussed relationship between openness and problematic smartphone use). Second, German study results help fill knowledge gaps due to proposed cross-cultural differences in personality (67). Third, in contrast to other literature that did not investigate other traits beyond neuroticism, the current paper explored all Big Five personality traits.

Results show that assessment of personality traits is of high importance for therapy. Patients with problematic smartphone use should be evaluated on neuroticism and extraversion. Therapeutic measures to lessen levels on these personality traits might also help in reducing smartphone-related problems. Also, the findings from this study suggest that clinicians should consider problematic smartphone use as a potential maladjustment related to psychopathology (depression and anxiety). Problematic smartphone use can be regarded as a dysfunctional strategy to regulate depressive emotions and feelings of loneliness. Psychotherapeutic interventions can support patients effectively in solving existing difficulties and problems.

## Data Availability Statement

The raw data supporting the conclusions of this article will be made available by the authors, without undue reservation.

## Ethics Statement

The studies involving human participants were reviewed and approved by Sigmund Freud University Vienna ethics committee. The patients/participants provided their written informed consent to participate in this study.

## Author Contributions

CE was involved in planning and supervising the work. AS processed the experimental data, performed the analysis, and drafted the manuscript. MS took the lead in writing the manuscript. All authors provided critical feedback and helped shape the research, analysis, and manuscript.

## Conflict of Interest

The authors declare that the research was conducted in the absence of any commercial or financial relationships that could be construed as a potential conflict of interest.
